# ECG gated 3D single shot FSE with variable TR for non-contrast peripheral MRA at 3T

**DOI:** 10.1186/1532-429X-17-S1-P413

**Published:** 2015-02-03

**Authors:** Xiangzhi Zhou, Cheng Ouyang, Aiming Lu, Mitsue Miyazaki

**Affiliations:** 1 Toshiba Medical Research Institute USA, Vernon Hills, IL, USA

## Background

ECG gated 3D single shot fast spin echo (SSFSE) sequence can be used to acquire systolic and diastolic data and the subtracted image gives the arterial blood signal. This technique, also called Fresh Blood Imaging (FBI), has been applied to image the peripheral artery without contrast infusion, in which each slice encoding (SE) step is acquired in a fixed TR (TR=n*RR). Ideally, TR should be sufficiently long for the recovery of the longitudinal magnetization. At 3T longer TR is preferred because of the increased blood T1 compare to the TR at 1.5T. Longer scan increases the uncomfortableness of the patient, and may introduce more motion artifacts. To reduce the scan time while still maintaining the blood signal, a new variable TR (vTR) method is proposed.

## Methods

**Figure 1:**
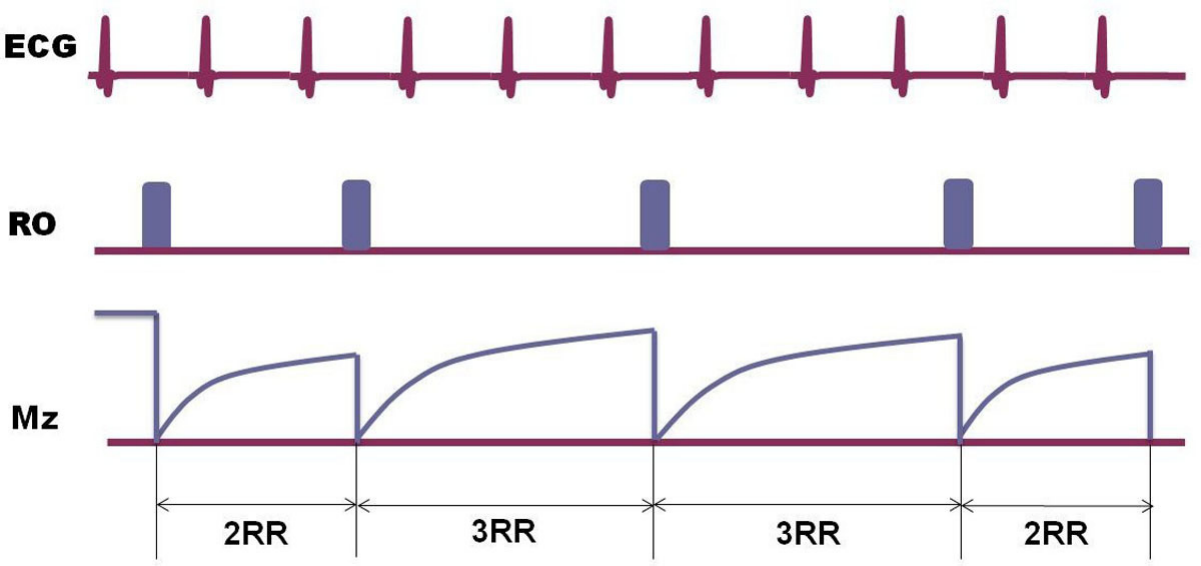
Demonstration of vTR FBI. Note in this example the TRs for the slice encoding at the k-space center are 3RRs while the rest TRs are spaced by 2RRs.

The study was approved by our institutional review board and informed consent was obtained. Five volunteers (29-60 years; 2 female) were enrolled and scanned by a Vantage Titan^TM^ 3T scanner (Toshiba Medical Systems Corporation, Otawara, Japan) equipped with Atlas SPEEDER^TM^ Spine coil and Atlas SPEEDER^TM^ Body coil. Followed by the localizer, pelvic, thigh and calf stations were imaged using FBI with ECG gating. The FBI sequence is modified to incorporate the vTR function, i.e., the slice encoding steps at the k-space center have longer TR and the rest have shorter TR (Figure 1). FBI parameters: 3D coronal SSFSE, TR=2-4RR with fixed TR, TR=2RR with vTR (2 extra RRs for the middle ~20% SE steps), TE=60ms, 80-100 slices, slice thickness=3mm, matrix 256X256; FOV 37cmX37cm, parallel imaging factor = 2, flip/flop angle=90°/140°, TDsys=0.22RR, TDdias=0.86RR, Resolution 1.4mmX1.4mm. Refine in RO, PE and SE directions. Overall image quality was blindly scored by 2 experienced clinical scientists (0: low, 4: high). Student t-test was performed to compare the image quality.

## Results

**Figure 2:**
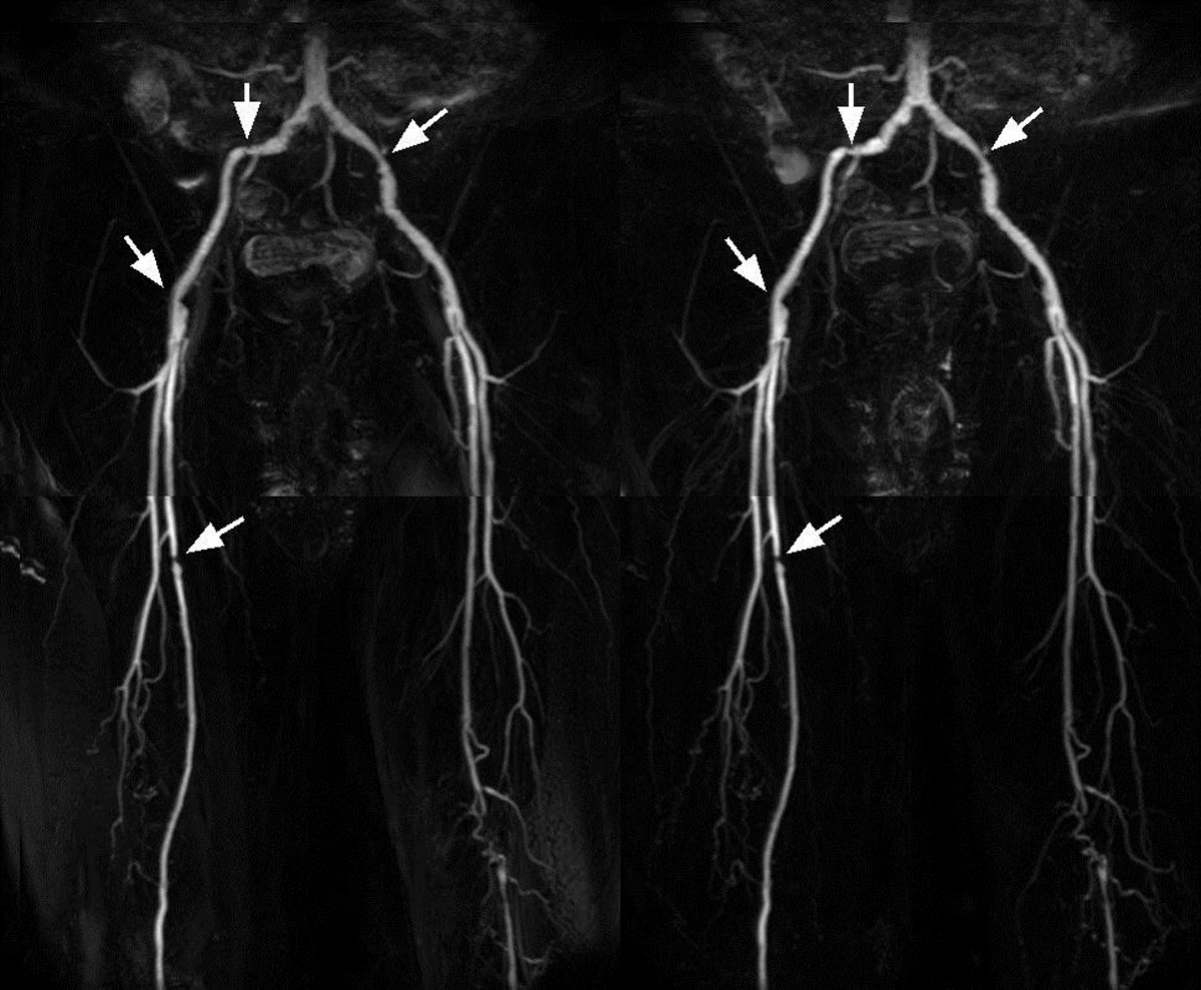
**Coronal MIP images of pelvic and thigh from a volunteer acquired by FBI with fixed TR (TR=4RR, left) and vTR (TR=2RR+20%4RR, right).** Note the stenosis-like narrower lumen (arrows) on the external iliac artery and right femoral artery can be identified in both images. The total scan times are 11:42 and 6:55 for each 2-station scan.

For fixed TR FBI, TR=2RR gives less arterial signal than longer TR. For TR=4RR and TR=2RR+20%4RR, the FBI coronal MIP images at the 3 stations showed comparable arterial image quality across all volunteers and the MIP image quality has no significantly difference (2.42±0.69 vs. 2.58±0.69, p=0.26). In terms of arterial signal intensity, the mixture feature of short and long TRs in vTR function offers a signal level between fixed long TR and short TR images, as expected. In the volunteer with narrower lumen on iliac arteries, FBI MIP images with and without vTR function can both clearly delineate the narrower lumen at different sites (Figure 2).

## Conclusions

The proposed vTR method offers significantly reduced scan time compare to fixed long TR FBI scan (20-40% scan time reduction). More advanced vTR patterns can be added with the purpose of reducing scan time while maintaining the arterial blood signal. One need to note that systolic and diastolic acquisition of FBI should have the same vTR setting to minimize the background tissue difference. More volunteer and patient data will be collected to further evaluate the performance of vTR FBI at 3T.

## Funding

N/A.

